# Lymphoma and Differentiated Thyroid Cancer: A Case Series

**DOI:** 10.7759/cureus.34429

**Published:** 2023-01-31

**Authors:** Amitha Sherief, Sugeeth M Thambi, Deepa Susan Joy Philip, Abhilash Menon, Sreekumar A

**Affiliations:** 1 Department of Medical Oncology, Regional Cancer Centre, Trivandrum, IND; 2 Department of Nuclear Medicine, Regional Cancer Centre, Trivandrum, IND

**Keywords:** chemotherapy, papillary carcinoma thyroid, non hodgkin's lymphoma, hodgkin lymphona, differentiated thyroid carcinoma

## Abstract

Occurrences of lymphoma and differentiated thyroid cancer are rare. Usually, involvement of the thyroid gland is seen as a part of extranodal involvement or as a part of radiation-induced malignant transformation in previously treated lymphoma patients. The incidence of synchronous hematological malignancy with differentiated thyroid cancer is 7%. The synchronous occurrence of differentiated thyroid cancer and lymphoma poses a significant diagnostic and treatment dilemma. Here we report a case series of four patients with lymphoma and differentiated thyroid cancer. All four patients had lymphoma treated first followed by definitive management of thyroid malignancy.

## Introduction

Differentiated thyroid cancer (DTC) is the most common endocrine malignancy, and among differentiated thyroid cancer, the most common is papillary carcinoma thyroid. The occurrence of DTC and hematological malignancy is very rare and constitutes an incidence of only 7% [1]. Literature review shows only very few cases of this entity and poses a diagnostic and treatment dilemma at times. Here we report four cases of lymphoma and DTC.

## Case presentation

Case 1

**Figure 1:**
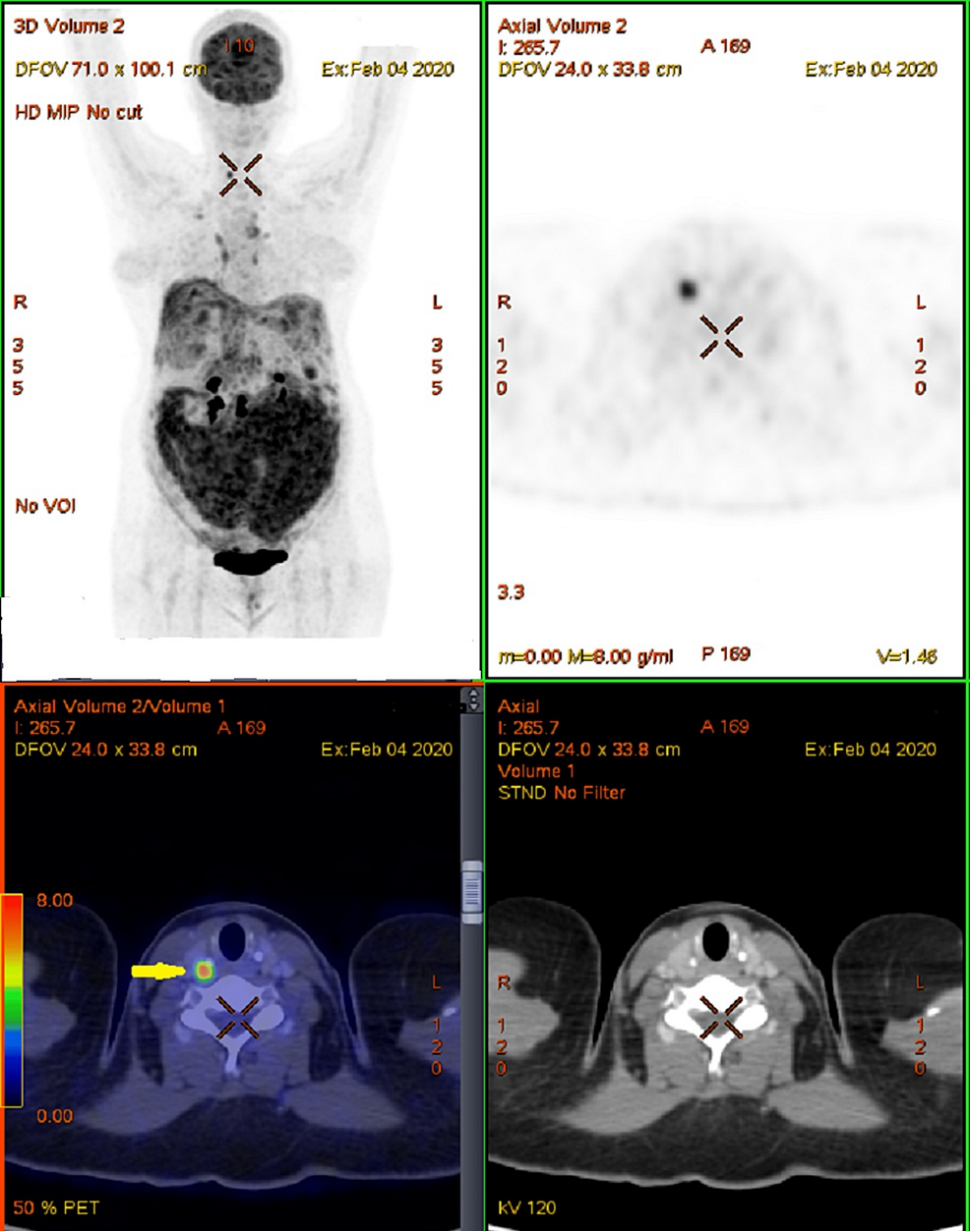
Pretreatment PET-CT images The images are showing supra and infra-diaphragmatic lymph nodes and focal uptake in the right thyroid (yellow arrow). PET-CT: positron emission tomography-computed tomography

A 41-year-old lady was evaluated for vague right-sided abdominal pain with gradually progressive abdominal distension of one-month duration with associated loss of weight and loss of appetite. The patient had an Eastern Cooperative Oncology Group performance status of one. On abdominal examination, she had gross ascites and multiple irregular palpable intraabdominal masses of varying size. An ultrasonogram (USG) of the abdomen and pelvis showed an ill-defined hypoechoic mass with necrotic areas 8x5 cm in the retro uterine space. An ultrasound-guided biopsy from the deposit showed atypical large cells arranged diffusely and infiltrating the adipose tissue with scanty vacuolated cytoplasm and irregular nuclear membrane. In immunohistochemistry study, these atypical cells were positive for CD20 and BCL6, and negative for CD5 and cytokeratin with a MIB1 labeling index of around 95%. The picture was compactable with diffuse large B cell lymphoma (DLBCL). At 8 months, the patient’s positron emission tomography and computed tomography (PET-CT) scan showed supra and infra-diaphragmatic lymph nodes with peritoneal, omental, perihepatic, mesenteric, splenic deposits and a hypodense lesion with focal uptake in the right lobe of thyroid (Stage IVBX) (Figure 1). Fine needle aspiration cytology (FNAC) from the thyroid lesion was suggestive of papillary thyroid carcinoma (PTC). The case was discussed in the multidisciplinary tumor board and she received combination chemotherapy with rituximab, cyclophosphamide, vincristine, adriamycin, and prednisolone (R-CHOP).

**Figure 2:**
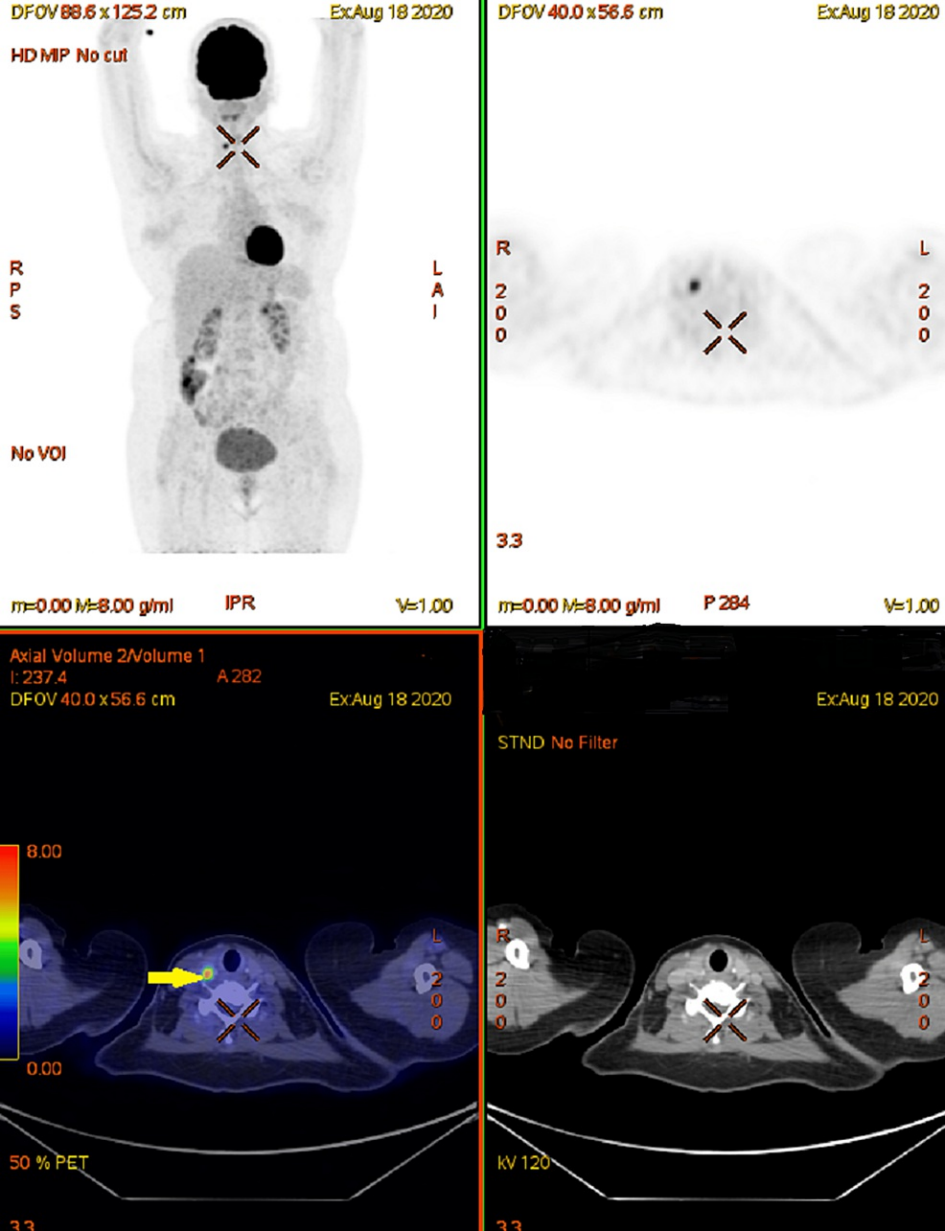
Post-chemotherapy PET-CT scan images The images are showing a complete metabolic response of lymph nodes with right thyroid uptake (yellow arrow).

Post six cycles of R-CHOP, the PET-CT scan was in complete metabolic remission with an FDG (fludeoxyglucose F18) non-avid mesenteric mass of 2 x 2 cm and a hypodense nodular lesion in the right lobe of the thyroid (Figure 2). The patient received consolidation radiotherapy (RT) to the mesenteric nodal mass, 30 Gray (Gy) in 15 fractions. Post-radiation, the patient underwent a total thyroidectomy. The histopathology was suggestive of papillary carcinoma thyroid pT1N0M0 (Stage I) and the patient was started on a suppressive dose of thyroxin and was kept on follow-up. After a disease-free interval of three months patient presented with abdominal pain, vomiting, and obstructive uropathy. A contrast-enhanced computed tomography (CECT) scan of the neck, thorax, abdomen pelvis was done which showed a right adnexal poorly defined solid cystic mass with calcification 6 x 5 cm and another mass in the left adnexa 8 x 6 cm extending to the pouch of Douglas (POD). USG-guided biopsy of the lesion was suggestive of DLBCL relapse. The patient was planned for salvage combination chemotherapy with rituximab, ifosfamide, carboplatin, and etoposide (R-ICE) for three-four cycles followed by reassessment for autologous stem cell transplant. Currently, the patient is undergoing the fourth cycle of R- ICE chemotherapy.

Case 2

A 37-year-old lady presented with an anterior neck swelling of two months duration. The patient was evaluated in a local hospital, underwent right hemi thyroidectomy and excision of right level II cervical lymph nodal swelling, and was referred to us for further management. Histopathological examination of the thyroid specimen was suggestive of papillary carcinoma of the thyroid and the right cervical lymph node was suggestive of DLBCL. The patient was evaluated with a PET-CT which showed a heterogeneous nodule in the left lobe of the thyroid with significant cervical and mediastinal nodes (Stage IIA). Bone marrow evaluation was negative for lymphoma involvement. She was planned for six cycles of chemotherapy with R-CHOP followed by completion of thyroidectomy. At three months, when she completed four cycles of R-CHOP chemotherapy, an interim CT scan was taken which showed near total resolution of cervical and mediastinal nodal mass with the left thyroid lobe showing a heterogenous nodule with an adjacent speck of calcification. She is continuing the planned course of chemotherapy.

Case 3

A 52-year-old lady was evaluated for right axillary swelling of three months duration. She underwent an axillary lymph node biopsy and was referred to us for further management. Histopathology examination of the specimen was suggestive of classical Hodgkin lymphoma (cHL). The patient was evaluated with a CECT scan of the neck, chest, abdomen, and pelvis which showed enlarged cervical, axillary, retroperitoneal, and inguinal lymph nodes and a subcentimetric nodule in the left lobe of the thyroid. Bone marrow evaluation was negative for lymphoma involvement. The patient was diagnosed to have stage IIIA cHL and planned for six cycles of combination chemotherapy with adriamycin, bleomycin, vinblastine, and dacarbazine (ABVD). Post three cycles of chemotherapy, an interim evaluation was done with CECT neck, chest, abdomen, and pelvis which showed a partial response with an additional new finding of subpleural fibrosis in both lung fields radiologically suggestive of bleomycin-induced lung toxicity. She received further chemotherapy as AVD (adriamycin, vinblastine, and dacarbazine) omitting bleomycin. At eight months, the end of treatment PET-CT scan showed complete remission with a 2 x 1.5 cm lesion in the left lobe of the thyroid. USG-guided FNAC from the thyroid lesion was suggestive of hurthle cell neoplasm. The patient underwent total thyroidectomy and the histopathology report was suggestive of hurthle cell carcinoma with a tumour size of 1.5 x 1.3 cm (stage I, pT1N0M0). The patient is now asymptomatic on thyroxin supplementation and is on regular follow-up at one year.

Case 4

A 29-year-old gentleman was evaluated for fever, drenching night sweats, and weight loss of three months duration. The patient was evaluated with a CECT scan of the neck, chest, abdomen, and pelvis which showed a bulky mediastinal mass of size 16 x 13 x 12.9 cm, multiple cervical nodes, a liver lesion, and a small nodule in the right lobe of the thyroid. Bone marrow evaluation was negative for lymphoma involvement. He underwent CT guided biopsy of the mediastinal mass which was suggestive of classical Hodgkin Lymphoma - nodular sclerosis type. The patient was diagnosed to have stage IVB cHL and planned for six cycles of ABVD. Post four cycles of chemotherapy, an interim evaluation was done with CECT neck, chest, abdomen, and pelvis which showed a partial response. At eight months, the PET-CT scan showed a complete metabolic response with a 0.9 x 0.7 cm lesion in the right lobe of the thyroid and a non-FDG avid mediastinal residual lesion of size 7.3 x 5.3 x 4.5 cm. He received IFRT 30Gy/15# for the residual mediastinal lesion. Meanwhile, he underwent USG-guided FNAC from the thyroid lesion which was suggestive of papillary carcinoma of the thyroid. The patient is planned for a total thyroidectomy.

Table 1 given below summarises all four patients in this case series.

**Table 1:** Summary of the four cases DLBCL: Diffuse Large B Cell Lymphoma, PTC - Papillary thyroid Carcinoma, cHL: classical Hodgkin's Lymphoma, R-CHOP: Rituximab, Cyclophosphamide, Adriamycin, Vincristine, Prednisolone, ABVD: Adriamycin, Bleomycin, Vinblastine, Dacarbazine, AVD: Adriamycin, Vinblastine, Dacarbazine, IFRT: Involved Field Radiation, DFS: Disease Free Survival, R-ICE: Rituximab, Ifosfamide, Carboplatin, Etoposide, NED: No Evidence of Disease.

Patient number	Case 1	Case 2	Case 3	Case 4
Age in years	41	37	52	29
Gender	Female	Female	Female	Male
Clinical presentation	Right-sided abdominal pain and abdominal mass	Swelling in the anterior aspect of the neck	Right axillary swelling	Cough, B symptoms
Diagnosis of Lymphoma	DLBCL, IVB	DLBCL, IIA	cHL, IIIA	cHL, IVB
Diagnosis of thyroid malignancy	PTC	PTC	Hurthle cell carcinoma thyroid	PTC
Treatment	R-CHOP X 6 + IFRT Followed by Total Thyroidectomy	R-CHOP X 6 followed by Completion Thyroidectomy	ABVD x 3 – AVD x 3 followed by Total Thyroidectomy	ABVD x 6 + IFRT followed by total thyroidectomy
Response to treatment	Metabolic complete response	On treatment	Metabolic complete response	Metabolic complete response
Status at last follow up	The patient had disease relapse (DFS 3 months), Now on salvage chemotherapy with R-ICE	On treatment	Alive NED at one year	Alive NED at 6 months

## Discussion

Differentiated thyroid cancer (DTC) is the most common endocrine malignancy [1]. Among DTCs, papillary carcinoma thyroid is the most prevalent thyroid cancer and is associated with a 20-year survival rate of more than 90% [2]. Synchronous occurrence of lymphoma and DTC is rare and constitutes only 7% of cases [1]. Patients with lymphoma who had neck irradiation can develop thyroid cancer as a late effect [3]. DLBCL is the most prevalent subtype of non-Hodgkin lymphoma. There are many studies that have reported the association of thyroid malignancy with lymphoma in those treated with radiotherapy [4]. Several studies attempted to explain the link between DTC and extra-thyroid malignancy implicating that “the long-term carcinogenic effects of specific cancer treatments might be responsible for second cancer” [1]. However, in our study four patients had lymphoma and thyroid malignancies both diagnosed over a short span of time and the most plausible explanation is the presence of yet unidentified molecular link or presence of general vulnerability carrying a higher risk for malignant transformation.

The increasing use of modern diagnostic imaging techniques has led to the identification of synchronous malignancies in a single person. The criteria for defining a tumour as a second primary neoplasm (SPN) were proposed by Warren and Gates in 1932 [5]. According to that, for a tumour to be called an SPN, it should have the following criteria met: a) each of the tumour should be malignancy confirmed by histology, b) there should be at least 2 cm of normal mucosa between the tumors and if the tumors are in the same location, then they should be separated in time by at least five years, c) The probability of one being the metastasis of other should be excluded. In our case series, the histological confirmation of lymphoma and thyroid malignancy was not done within a period of six months, so the synchronous nature of the malignancy could not be ascertained.

A case series from China reported eight cases of synchronous lymphoma and papillary thyroid cancer. All patients were females and had papillary carcinoma thyroid. Five of them had DLBCL and one case each of Hodgkin lymphoma, MALT lymphoma, and Peripheral T cell lymphoma [6]. Our case series had two cases of DLBCL and HL each. A study by Al Saidan et al. describes a case of synchronous lymphoma and thyroid cancer in a male patient [7]. A study from Iraq reported a case of synchronous lymphoma and Hurthle cell carcinoma in 2021, similar to our study [8].

The synchronous occurrence of lymphoma and DTC is both a diagnostic and therapeutic challenge. The importance of the lymphoma-first approach is highlighted by Dhanani et al. and Popivanov et al. [9,10]. According to them, PTC is the most idle form of thyroid cancer, and enlarged cervical lymphadenopathy secondary to lymphoma will disappear or regress after the treatment of lymphoma, thus making surgery for PTC much easier with fewer complications. Thus we also started the chemotherapy for lymphoma first in our patients.

## Conclusions

Occurrences of lymphoma and differentiated thyroid malignancy together are rare. The use of modern diagnostic imaging techniques has helped in identifying the same. Usually, lymphomas are treated first followed by management of the thyroid malignancy. The treatment of synchronous lymphoma and thyroid malignancies is often challenging. Even though in our case series the histological confirmation of both lymphoma and thyroid malignancies was not done within a period of six months, all patients had radiological evidence of the same at the initial workup itself. Often individualized treatment decisions are required. All our patients had the lymphoma-first approach and had an excellent prognosis. Further studies are required to find out common molecular abnormalities responsible for the synchronous occurrence of both lymphoma and thyroid malignancies.
